# Isolation and characterization of *Stenotrophomonas pavanii* GXUN74707 with efficient flocculation performance and application in wastewater treatment

**DOI:** 10.3389/fmicb.2024.1367043

**Published:** 2024-04-26

**Authors:** Siqi Qin, Qiao Li, Jin Dou, Yuanyuan Man, Liuyan Wu, Hongmei Tian, Mingguo Jiang, Guofang Liu

**Affiliations:** Guangxi Key Laboratory for Polysaccharide Materials and Modifications, School of Marine Sciences and Biotechnology, Guangxi Minzu University, Nanning, China

**Keywords:** bioflocculants, *Stenotrophomonas pavanii*, flocculation performance, decolorization, wastewater

## Abstract

The identification of microorganisms with excellent flocculants-producing capability and optimization of the fermentation process are necessary for the wide-scale application of bioflocculants. Therefore, we isolated and identified a highly efficient flocculation performance strain of *Stenotrophomonas pavanii* GXUN74707 from the sludge. The optimal fermentation and flocculation conditions of strain *S. pavanii* GXUN74707 was in fermentation medium with glucose and urea as the carbon and nitrogen sources, respectively, at pH 7.0 for 36 h, which treatment of kaolin suspension with 0.5 mL of the fermentation broth resulted in a flocculation rate of 99.0%. The bioflocculant synthesized by strain *S. pavanii* GXUN74707 was found mainly in the supernatant of the fermentation broth. Chemical analysis revealed that the pure bioflocculant consisted of 79.70% carbohydrates and 14.38% proteins. The monosaccharide components of MBF-GXUN74707 are mainly mannose (5.96 μg/mg), galactose (1.86 μg/mg), and glucose (1.73 μg/mg). Infrared spectrometric analysis showed the presence of carboxyl (COO-), hydroxyl (-OH) groups. The SEM images showed clumps of rod-shaped bacteria with adhesion of extracellular products. Furthermore, the strain decolored dye wastewater containing direct black, direct blue, and Congo red by 89.2%, 95.1%, 94.1%, respectively. The chemical oxygen demand (COD) and biological oxygen demand (BOD) removal rates after treatment of aquaculture wastewater with the fermentation broth were 68% and 23%, respectively. This study is the first to report the performance and application of strain *Stenotrophomonas pavanii* in wastewater flocculation. The results indicate that strain *S. pavanii* is a good candidate for the production novel bioflocculants and demonstrates its potential industrial practicality in biotechnology processes.

## 1 Introduction

Flocculation is a simple, low-cost, and environmentally friendly process that can separate and remove suspended particles, particulate dyes, and heavy metals from various types of wastewaters (Zhao et al., 2017; Wang et al., 2020). There are three main types of flocculants, including inorganic, synthetic organic, and biological flocculants (Aljuboori et al., 2015; Pu et al., 2018). Inorganic flocculants and synthetic organic flocculants are widely used in industry due to their efficiency and cost-effectiveness (Nontembiso et al., 2011). However, this widespread use generates large amounts of waste, which is difficult to degrade, leading to secondary pollution and various environmental and health-associated problems (Aljuboori et al., 2013; Ayangbenro et al., 2019).

Bioflocculants have attracted extensive research and application as alternatives due to their biodegradability, as well as having the advantages of being environmentally friendly, non-toxic, and effective (Liu et al., 2009; Chopra and Ruhi, 2016). Microbial flocculants (MBF) are a cost-effective and practical solution for removing environmental pollutants.

The use of biological flocculants to remove dyes from industrial wastewater seems promising, but there are still gaps in research. Currently, the large-scale production and application of bioflocculants are limited by factors such as high production costs, long fermentation cycles, and poor practical applicability (Aljuboori et al., 2014). The discovery of microorganisms with better flocculation ability is thus an urgent problem to be solved. To date, the most effective strategies for improving production and flocculation activity have involved modifications of the culture medium composition and fermentation conditions (Guo and Chen, 2017; Rasulov et al., 2017). Another factor that has contributed significantly to the poor development of bioflocculants is a lack of bacterial resources. Thus, the identification of flocculants-producing bacteria is an important research focus.

Developments in dye production and technology have led to sharp increases in the discharge of wastewater from the dye industry. Dye wastewater is associated with a series of problems such as its high chromaticity, compositional complexity, high biological toxicity, and poor biochemical degradation, presenting significant challenges to its treatment (Correia et al., 1994). The heavy metals, chlorinated compounds, pigments and other pollutants in textile wastewater are considered the main culprits of water pollution, which can lead to oxidative stress, reduce photosynthesis and CO_2_ assimilation rates, and thus reduce plant growth (Naresh Yadav et al., 2020; Pradeep Kumar et al., 2021). Dye wastewater contains a variety of organic pollutants, which consume dissolved oxygen in the water and thus disrupt the ecological balance of the water. At the same time, wastewater also has a major impact on human health and safety (Liu et al., 2018). Currently, physical, chemical, and biological methods can be used for the decolorization and degradation of the components of dye wastewater. In terms of biological treatment, flocculation is key (Durmaz and Sanin, 2001; Yang et al., 2013; Manavi et al., 2017).

At present, the hot topics in wastewater treatment strategies are nanoparticles and the genetically engineered bacteria. Nanoparticle processing has high efficiency but high cost. Genetic engineering bacteria selectively modify related enzymes to enhance their ability to remediate wastewater (Manikant et al., 2023; Tripathi et al., 2023). Scholars have successfully used microorganisms such as bacteria, fungi, yeast, and algae for dye remediation. Microorganisms can break the azo bonds present in dye molecules through their enzymatic activity (Shweta and Manikant, 2017; Sharma and Qanungo, 2022). The process cost of microbial treatment of wastewater is low, while also ensuring environmental issues and eliminating the need for secondary treatment. Among them, bacteria have extremely strong survival ability, can adapt to various extremely harsh environments, and their decolorization ability is also extremely significant. Bacteria become an ideal choice for wastewater treatment. There are many types of flocculants producing bacteria, and literature reports that *Oerskovia paurometabola* (Rita Dias Guardão et al., 2020), *Brevundimonas diminuta* (Saravanan et al., 2022), *Geobacillus thermoleovorans* (Julián et al., 2020), *Serratia* spp. (Li-zhong et al., 2023), *Lysinibacillus sphaericus* (Shantkriti et al., 2022) have outstanding abilities in treating dye decolorization.

In the present study, strain *Stenotrophomonas pavanii* GXUN74707 was isolated and identified from sludge. The strain showed highly efficient flocculation and dye decolorization activities. This study is the first to report that *S. pavanii* can produce bioflocculant, and the findings indicate the significant potential of the strain in wastewater flocculation and dye decolorization.

## 2 Materials and methods

### 2.1 Bacterial strains and media

Strain *S. pavanii* GXUN74707 was isolated from the sludge in Guangxi Minzu University and has been deposited in the China General Microbiological Culture Collection Center (CGMCC No. 27475). The activation culture of the strain was LB medium containing 10 g/L of tryptone, 5 g/L of yeast extract, and 10 g/L of NaCl, with the pH adjusted to 7.0–7.5. The fermentation medium contained 10 g/L of glucose, 0.5 g/L of yeast extract, 0.5 g/L of urea, 5 g/L of K_2_HPO_4_, 2 g/L of KH_2_PO_4_, 0.1 g/L of NaCl, and 2 g/L of MgSO_4_ ⋅ 7H_2_O, pH 7.0–7.5.

### 2.2 Identification of strain GXUN74707

The 16S rRNA gene sequence was analyzed to identify the strain GXUN74707. Genomic DNA was extracted from the target strain and was amplified by PCR using the universal primers 27F/1541R for the 16S rRNA gene. The 16S rRNA gene sequence was compared with sequences using BLAST on the EzBioCloud website (http://www.ezbiocloud.net/). The aligned sequence was analyzed using MEGA 11.0 software (Batta et al., 2013).

### 2.3 Determination of flocculation activity

The strain GXUN74707 was cultured in flasks containing 30 mL LB medium at 30°C with shaking at 180 rpm until the OD_600_ = 1.0. It was then transferred to the fermentation medium at an inoculation rate of 1%, and the flocculation rate of the fermentation broth was determined. Strains with higher flocculation activity were selected for further investigation. The flocculation activity was measured using a kaolin clay suspension (0.25 g of kaolin clay in 50 mL distilled water). The kaolin suspension was mixed with 1.5 mL fermentation broth, and 5 mL of 1% CaCl_2_ was added to the mixture. The pH of the kaolin suspension was adjusted to 7.0–7.5 with NaOH. The mixture was vortexed for 2 min and left standing for 5 min. The optical density of the supernatant against a blank (distilled water) was measured at 550 nm. The flocculation rate was defined as follows:

$$E\left(\%\right)=\frac{B-A}{B}*100\%$$

where E is the flocculation rate (%), A is the absorbance of the kaolin suspension processed by the sample, and B is the absorbance of the control (blank kaolin suspension) (Batta et al., 2013).

### 2.4 Optimization of cultivation conditions

Optimization of fermentation time: Strain GXUN74707 was inoculated into LB medium and cultured until OD_600_ = 1.0. It was then transferred to the fermentation medium at a 1% inoculation rate, and fermentation was continued at 30°C and 180 rpm for 72 h. The flocculation rate of the fermentation broth was measured every 12 h.

Optimization of initial pH: The pH of the fermentation medium was adjusted with 1 mol/L of NaOH or HCl to a pH range of 4.0–10.0. The strain GXUN74707 was transferred to fermentation media with different pH values, and fermentation was continued at 30°C and 180 rpm for 36 h. The flocculation rate of the strain fermentation broth under different pH conditions was measured.

Nitrogen source optimization: Yeast extract, casein, peptone, urea, beef extract, tryptone, and (NH_4_)_2_SO_4_ were used as nitrogen sources for fermentation. Strain GXUN74707 was cultured for 36 h in media containing different nitrogen sources. The flocculation rates were measured with kaolin suspension and compared to determine the optimum nitrogen source.

Carbon source optimization: Glucose, maltose, sucrose, fructose, galactose, starch, and sodium acetate were used as carbon sources. Strain GXUN74707 was cultured for 36 h in media containing different carbon sources. The flocculation rates were measured with kaolin suspension and compared to determine the optimum carbon source.

### 2.5 Extraction and purification of bioflocculant

The fermentation broth was centrifuged at 8,000 rpm for 10 min to precipitate the bacteria. The supernatant was then mixed with 2 volumes of cold ethanol and left to stand at 4°C overnight. The resultant precipitate was collected by centrifugation at 8,000 rpm for 15 min, yielding the crude bioflocculant. The crude bioflocculant was dissolved in distilled water at a ratio of 1:4 (v/v) and lyophilized (Choi et al., 1998; Subudhi et al., 2014).

### 2.6 Characteristics of MBF-GXUN74707

Distribution of microbial flocculant: The fermentation broth was centrifuged, which the bacteria and supernatant were collected. The bacteria were resuspended in distilled water to generate to cell content sample. The kaolin suspension was treated with equal volumes of fermentation broth (containing bacterial bodies), supernatant, and resuspension of bacterial fluid, and the flocculation rate was measured.

Chemical composition: The total sugar content and protein content of the microbial flocculant was determined by the phenol-sulfuric acid method using glucose as the standard solution (Chaplin and Kennedy, 1986) and the Bradlord method using bovine serum albumin as the standard solution (Marion, 1976), respectively.

Determination of monosaccharides composition: The monosaccharides composition of MBF-GXUN74707 were determined by ion chromatography (ThermoFisher, ICS5000) using Dionex ICS-5000+ system, Carbo PACTM PA20 (2.0 mm × 150 mm) anion exchange column as analysis column, electrochemical detector, and gradient elution method. The monosaccharides composition of MBF- GXUN74707 were analyzed.

Fourier-transform infrared spectroscopy (FTIR) of the MBF-GXUN74707: The functional groups of the bioflocculant were determined using an FTIR spectrophotometer (Thermo Electron Corporation, Waltham, MA, USA) over a wavenumber range of 4,000–400 cm^–1^ (Gomaa, 2012).

Scanning electron microscopy (SEM): A Supra 55 Sapphire microscope (Zeiss, Germany) was used to evaluate the bacterial strain (Chen et al., 2017).

### 2.7 Investigation of the flocculation process

To assess the flocculation properties of MBF-GXUN74707, the effects of different fermentation broth dosage and times on the measurement of flocculation by the kaolin suspension were determined. Different volumes of fermentation broth (0.2, 0.5, 1, 1.5, 2, 2.5, and 3 mL) to a 5 g/L kaolin suspension and the flocculation rates were measured. To assess the times, the kaolin suspension was allowed to stand for different times (1, 2.5, 5, 7.5, 10, and 15 min) before measuring the flocculation rate. The comparison of the flocculation rates allowed the determination of the optimal flocculation conditions for the microbial flocculant produced by strain *S. pavanii* GXUN74707.

### 2.8 Dye decolorization

2 mL of fermentation broth containing strain *S. pavanii* GXUN74707 were added to 5 mL of 1% CaCl_2_, and 93 mL of dye solution (50 mg/L) in a 500 mL beaker, and the pH was adjusted using NaOH. The solution was mixed rapidly (200 rpm) for 1 min, followed by slow mixing (100 rpm) for 2 min, after which it was transferred to a 100 mL measuring cylinder and allowed to sediment for 60 min. After flocculation, the supernatants were collected at 1 cm below the wastewater surface and the absorbance at the maximum absorption wavelength of the dye was measured using a UV spectrophotometer. The dye removal rate was calculated as follows:

$$M\left(\%\right)=\frac{m-n}{m}*100\%$$

Where M is the dye decolorization rate, and m and n are the initial absorbances of the dye and the absorbance after treatment, respectively (Chen et al., 2017; Pathak et al., 2017).

### 2.9 Removal of COD and BOD from aquaculture wastewater

The aquaculture wastewater samples were obtained from fish-farming wastewater in a laboratory. Changes in the chemical oxygen demand (COD) and biological oxygen demand (BOD) before and after the treatment of the fish-farming wastewater with fermentation broth of strain *S. pavanii* GXUN74707 were measured. The COD using the dichromate method and BOD was measured using the dilution inoculation method (Prasertsan et al., 2006; Pathak et al., 2017). The samples were sent to Guangxi Nanhuan Testing Company for testing.

## 3 Results and discussion

### 3.1 Isolation and identification of flocculants-producing bacteria

A total of 33 strains of bioflocculants-producing bacteria were isolated from the sludge in Guangxi Minzu University. Of these, the strain named GXUN74707 which showed the highest flocculation activity (82.3%) was selected for further study. Strain GXUN74707 has been deposited in the China General Microbiological Culture Center (CGMCC No. 27475).

**FIGURE 1:**
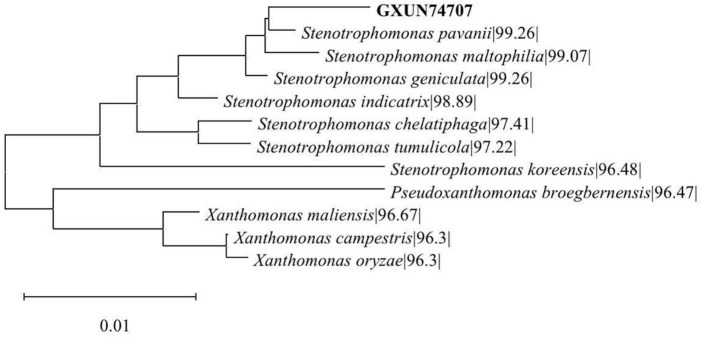
GXUN74707 phylogenetic tree. The phylogenetic tree was constructed using Mega 11.

Genomic DNA was extracted from stain GXUN74707, and the 16S rRNA gene was amplified by PCR. The PCR product was analyzed by agarose gel electrophoresis, which showed the expected size (1,500 bp). The nucleotide sequence was compared using the EzBioCloud website, and BLAST analysis of the nucleotide sequence showed strain GXUN74707 has 99.26% similarity to the *Stenotrophomonas pavanii*. A phylogenetic tree (Figure 1) was constructed using the N-J method to identify the 16S rRNA gene sequence of the strain. The results showed that the strain had the closest genetic relationship with the *Stenotrophomonas pavanii*, and was named *S. pavanii* GXUN74707.

### 3.2 Optimization of fermentation and flocculation conditions for bioflocculant production

#### 3.2.1 The effect of fermentation time on bioflocculant production

**FIGURE 2:**
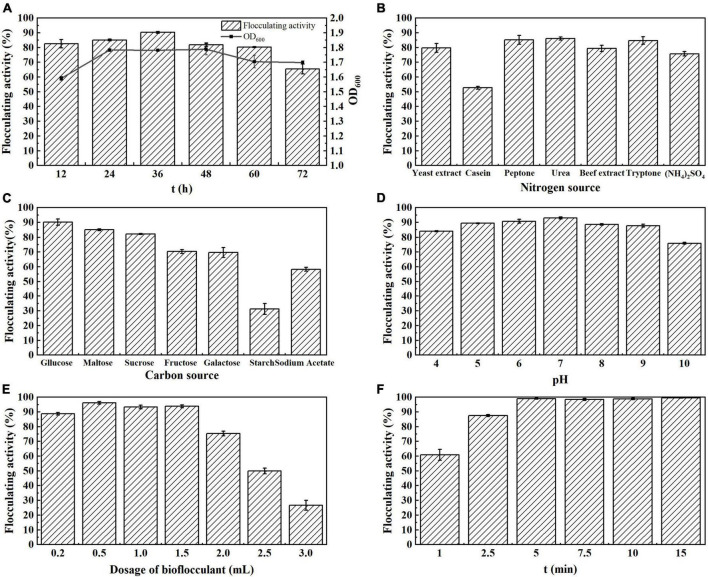
Flocculation properties of MBF-GXUN74707. **(A)** Effect of fermentation time; **(B)** effect of nitrogen source; **(C)** effect of carbon source; **(D)** effect of initial pH; **(E)** effect of fermentation broth dosage; **(F)** effect of standing time. Data are mean ± standard deviations from three replicates.

The growth rate of strain *S. pavanii* GXUN74707 showed a parallel relationship with the flocculation activity. The flocculation rate was highest during the stable period (36 h). As the culture time increased, the flocculation activity decreased slightly and then stabilized (Figure 2A). The results indicated that the flocculation rate of strain *S. pavanii* GXUN74707 was related to its growth and increased in synchrony with the growth of the strain.

#### 3.2.2 The effect of nitrogen source on bioflocculant production

Nitrogen sources are extremely important for bioflocculant production and play a crucial role in cell growth and synthesis (Rasulov et al., 2017). The effect of the nitrogen source on bioflocculant production was investigated, finding that peptone, urea, and tryptone all enhanced the flocculation activity of strain *S. pavanii* GXUN74707, with a flocculation rate of over 80%. Among them, when urea was used as the nitrogen source, the flocculation rate reached 86.2% (Figure 2B). Urea was thus selected as the optimal nitrogen source in subsequent experiments because of its favorable effects on bioflocculant production.

#### 3.2.3 The effect of carbon source on bioflocculant production

The synthesis of bioflocculant is affected significantly by the type of carbon source. The effects of various single carbon sources, including glucose, maltose, sucrose, fructose, galactose, starch, and sodium acetate, on bioflocculant synthesis by strain *S. pavanii* GXUN74707 were evaluated. As shown in Figure 2C, flocculation rate reached 90.4% when glucose was used as the carbon source. In contrast, when starch was used as the carbon source in the medium, the flocculation rate was only 31.3%. This indicated that glucose was the optimal carbon source for the fermentation culture of strain *S. pavanii* GXUN74707, and was conducive to the synthesis of bioflocculant.

#### 3.2.4 The effect of initial pH on bioflocculant production

The initial pH of the fermentation medium had a direct influence on bioflocculant synthesis. The initial pH of the medium was adjusted using NaOH or HCl solutions and the effects of different pH over the range of 4.0–10.0 on flocculation rate were analyzed. It was found that strain *S. pavanii* GXUN74707 could produce flocculants over a range of initial pH values, with the flocculation rate remaining above 80% (Figure 2D). These results indicated that the strain could produce bioflocculant in acidic, neutral, and alkaline environments, with a wide range of applications, suggesting that it could adapt to different environments and maintain flocculation activity, even surviving in strongly acidic and alkaline environments while synthesizing bioflocculant (Nakata and Kurane, 1999; Xia et al., 2008).

#### 3.2.5 The effect of fermentation broth dosage on bioflocculant production

It was found that the flocculation rate of strain *S. pavanii* GXUN74707 was best when the dosage of the fermentation broth was 0.5 mL. As the dosage of the fermentation broth increased, especially when it exceeded 1.5 mL, the flocculation rate decreased significantly (Figure 2E). This indicated that flocculation was dependent on the optimal bacterial concentration. This suggested that when the bioflocculant concentration was insufficient, the probability of particle collision in the system decreases, which affected the flocculation rate. In contrast, the addition of excessive bioflocculant was likely to alter the charge of the colloidal system, reducing the flocculation effect (Gong et al., 2008).

#### 3.2.6 The effect of standing time on bioflocculant production

Bioflocculant can accelerate the collision, coagulation, and sedimentation of colloidal particles in a solution. A short standing time can lead to insufficient sedimentation, resulting in poor flocculation effects, while long standing times will increase the duration of treatment and economic costs. It was found that after treatment of the kaolin suspension with the fermentation broth of strain *S. pavanii* GXUN74707, the flocculation rate reached 99.0% when allowed to stand for 5 min. No significant changes in the flocculation rate were seen after standing for more than 5 min (Figure 2F).

### 3.3 Characterization of the bioflocculant

#### 3.3.1 Distribution of bioflocculant

**FIGURE 3:**
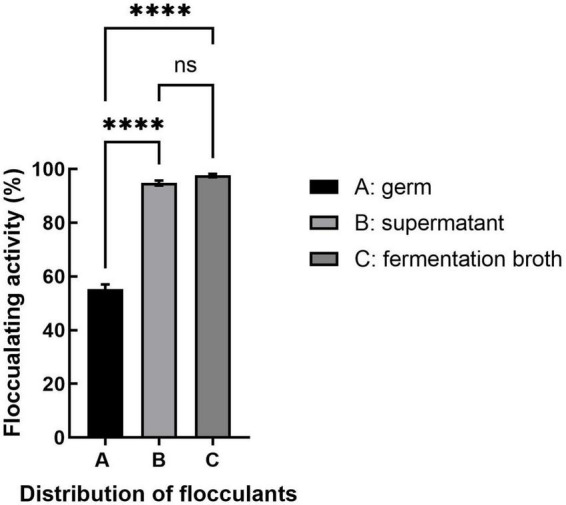
Distribution of flocculation activity. The bacterial fermentation broth was centrifuged and kaolin suspensions were treated with equal volumes of fermentation broth (containing bacterial cells), supernatant, and strain resuspension, followed by measurement of the flocculation rate. “ns” stands for *P* > 0.05; “^*⁣*⁣**^” stands for *P* < 0.001.

Bioflocculants produced by different microorganisms vary in their distribution, with some attached to the cell surface and others dissociated from the fermentation broth, which will affect their purification. As shown in Figure 3, the bioflocculant produced by strain *S. pavanii* GXUN74707 was found mainly in the supernatant of the fermentation broth, while the flocculant rate of the bacterial body itself was only 55.3%. It appeared that the bioflocculant secreted by strain *S. pavanii* GXUN74707 was a metabolic product synthesized and secreted into the extracellular space during bacterial growth. The extraction of the bioflocculant could be achieved through ethanol precipitation of the fermentation supernatant.

#### 3.3.2 Chemical composition of bioflocculant

In order to understand the flocculation mechanism of bioflocculant, it is necessary to determine its composition. This will help to improve flocculation parameters and improve performance during the application. In this study, the chemical analysis of MBF-GXUN74707 was carried out to determine the composition of total sugars, monosaccharides and proteins. The results of phenol-sulfuric acid analysis showed that the bioflocculant contained 79.70% total sugar, and the results of Bradford analysis showed that the bioflocculant contained 14.38% protein. The results of ion chromatography analysis showed that the monosaccharides composition of the MBF-GXUN74707 was mainly composed of mannose (5.96 μg/mg), galactose (1.86 μg/mg), glucose (1.73 μg/mg), etc (Table 1). The analysis of chemical composition showed that the active component of the microbial flocculant was mainly sugar, in addition, the high sugar content confirmed our previous research that the biological flocculant was heat stable. While bioflocculants based on protein content are heat sensitive because proteins are easily denatured at high temperatures.

**TABLE 1:** Monosaccharides composition of MBF-GXUN74707.

Name	μg/mg
Mannose	5.96
Galactose	1.86
Glucose	1.73
Rhamnose	1.16
Fucose	0.69
Glucosamine hydrochloride	0.37
Glucuronic acid	0.34
Ribose	0.20
Arabinose	0.06

#### 3.3.3 Infrared spectrophotometry (FTIR)

**FIGURE 4:**
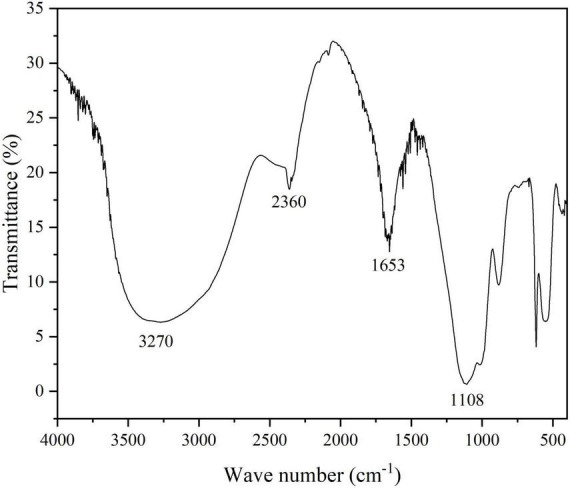
Fourier-transform infrared spectroscopy. Characterization of MBF-GXUN74707.

Infrared spectrophotometry was used to analyze MBF-GXUN74707 as illustrated in Figure 4. Clear absorption peaks were observed at 3,270, 2,360, 1,653, and 1,108 cm^–1^, indicating the presence of characteristic polysaccharide groups. The peak at 3,270 cm^–1^ was broad, and was attributed to -OH stretching vibrations in the sugar ring, while the small absorption peak at 2,360 cm^–1^ resulted from C-H stretching vibrations, the peak at 1,653 cm^–1^ reflected C = O asymmetric stretching vibrations in -COO-, and the 1,108 cm^–1^ peak was the characteristic C = O absorption peak of ether in the polysaccharide ring. The presence of these absorption peaks was typical of carbohydrate derivatives and the presence of these functional groups indicates the effectiveness of strain *S. pavanii* GXUN74707 as a flocculating agent (Xia et al., 2022; Yilmaz et al., 2022).

#### 3.3.4 Scanning electron microscopy

**FIGURE 5:**
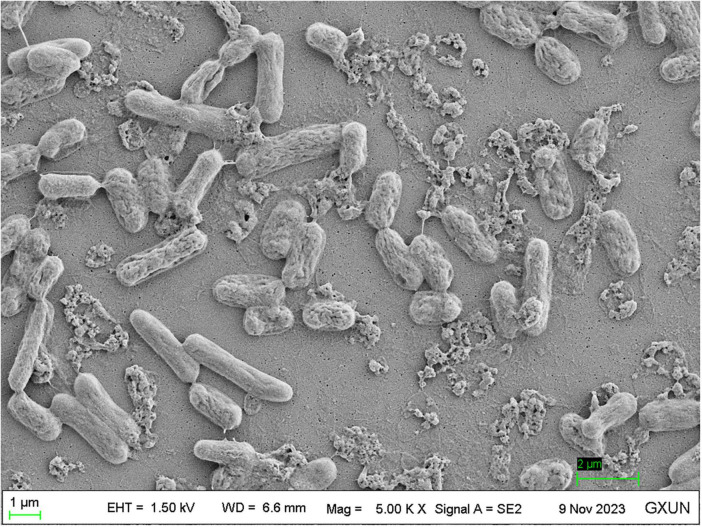
Scanning electron microscopy image of strain *S. pavanii* GXUN74707. Microbial flocculant was distributed near the bacterial bodies.

The SEM evaluation of strain *S. pavanii* GXUN74707 showed the surface morphology of the bacterial body with the production of extracellular bioflocculant (Figure 5; Li et al., 2008; Raveendran et al., 2013). The SEM images showed clumps of rod-shaped bacteria with adhesion of extracellular products, most probably the bioflocculant.

### 3.4 Application of the bioflocculant in dye wastewater and aquaculture wastewater

#### 3.4.1 Decolorization by bioflocculant in dye removal

**FIGURE 6:**
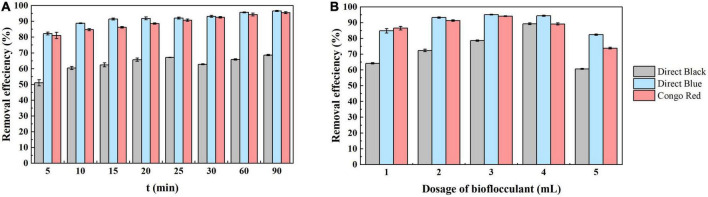
Microbial flocculant has great application value in dye decolorization. Add the fermentation broth of the strain *S. pavanii* GXUN74707 to three different dye solutions (including direct black, direct blue, and Congo red dyes, respectively). And observe the effects of different factors on dye decolorization **(A)** settling time; **(B)** fermentation broth dosage. Data are mean ± standard deviations from three replicates.

The viscosity and adsorption properties of polysaccharides can enhance the aggregation of dye particles to form larger flocs after addition of the bioflocculant to dye wastewater (Saratale et al., 2011). Here, the application of bioflocculant produced by strain *S. pavanii* GXUN74707 in the decolorization of dye wastewater was assessed by observation of the decolorization of different solutions containing commonly used dyes (direct black, direct blue, and Congo red) by the fermentation broth of strain *S. pavanii* GXUN74707 (Somasundaran and Runkana, 2005; Chouchane, 2018). First, the settling time after bioflocculant addition was investigated. After the addition of the bioflocculant, the three dye solutions showed rapid adsorption and decolorization. After 5 min of standing, the decolorization rates of the solutions containing direct black, direct blue, and Congo red were 51.1%, 82.2%, and 81.1%, respectively. The results provided compelling evidence that the adsorption of dye by the bioflocculant was a rapid process, although some particles remained suspended in the solution (Chen et al., 2017). With the extension of the settling time, the dye particles were essentially completely settled and after 90 min, the decolorization rate of direct black dye was 68.6%, that of direct blue was 96.6% and that of Congo red was 95.5% (Figure 6A).

In addition, the concentration of bioflocculant can affect decolorization (Wu et al., 2012). As the bioflocculant concentration increases, the availability of adsorption surfaces and sites increases, leading to an increase in the decolorization rate. After the addition of varying doses of fermentation broth (1–5 mL) to 100 mL of dye solution, the results (Figure 6B) showed that the optimal dosage for the fermentation broth of strain *S. pavanii* GXUN74707 to adsorb direct black, direct blue, and Congo red dyes is 4 mL (89.2%), 3 mL (95.1%), and 3 mL (94.1%), respectively. Further increasing the dosage of the adsorbent will lead to a slight decrease in adsorption efficiency. It is possible that excessive amounts of bioflocculant might lead to imbalances in the charge state within the solution, which was not conducive to collisions between particles, inhibited the formation of small flocs, and thus reduced the decolorization rate.

#### 3.4.2 Application of bioflocculant to aquaculture wastewater

In China and many other areas with large populations, there has been a rapid development of aquaculture (Boyd et al., 2022; Wu et al., 2022). The potential applications of strain *S. pavanii* GXUN74707 in aquaculture wastewater were evaluated by determining the COD and BOD concentrations. The initial concentrations of COD and BOD in aquaculture wastewater were 421 mg/mL and 3 mg/mL, respectively. After treatment of the wastewater with strain *S. pavanii* GXUN74707 fermentation broth, the COD concentration decreased to 134 mg/mL, with a removal rate of 68%, while the BOD concentration decreased to 2.3 mg/mL with a removal rate of 23% (Table 2).

**TABLE 2:** Removal efficiency of COD and BOD in aquaculture wastewater treated with *S. pavanii* GXUN74707.

Water quality	COD	BOD
Before treatment (mg/mL)	421	3
After treatment (mg/mL)	134	2.3
Removed efficiency (%)	68%	23%

According to China’s sewage discharge standards, the first-level standard of COD is 100 mg/mL, the second-level standard is 150 mg/mL, and the third-level standard is 500 mg/mL. The first-level standard of BOD is 30 mg/mL, the second-level standard is 60 mg/mL and the third-level standard is 300 mg/mL. The aquaculture wastewater treated with strain *S. pavanii* GXUN74707 meets the national level 2 discharge standard for COD and Level 1 discharge standard for BOD. The results indicated that strain *S. pavanii* GXUN74707 was effective for the removal of both COD and BOD from aquaculture wastewater (Comte et al., 2006).

## 4 Conclusion

Strain *Stenotrophomonas pavanii* GXUN74707 was isolated from sludge and was found to have excellent flocculation performance. The optimal culture conditions were determined and treatment of kaolin suspension indicated a maximum flocculation rate of 99.0%. The study also found that strain *S. pavanii* GXUN74707 had excellent decolorization performance, showing 89.2, 95.1, and 94.1% decolorization of dye wastewater containing direct black, direct blue, and Congo red, respectively. Treatment of aquaculture wastewater with the fermentation broth of the strain resulted in the removal by 68% of COD and 23% of BOD.

Although bioflocculants are promising substitutes for chemical flocculants, their high cost and low production efficiencies have limited their industrial application. Here, it was found that the strain *S. pavanii* GXUN74707 could be cultured in simple and easily obtained medium with a fermentation temperature of 30°C, pH of 7.0, and fermentation time of 36 h. As shown by the results, the fermentation conditions are mild and a large amount of fermentation broth with efficient flocculation performance can be obtained in a short period of time. The bioflocculant production of the strain was determined under laboratory conditions, indicating a simple production process, high yield, stable properties, and easy preservation, suggesting its potential in large-scale production and application.

Strain *S. pavanii* GXUN74707 has also been found to have significant potential for sewage treatment. The present study find that strain *S. pavanii* GXUN74707 shows good flocculation and decolorization activity in a variety of environments. Next, we will combine national standards to detect the situation of wastewater treated with strain *S. pavanii* GXUN74707, and apply molecular biology and bioinformatics to explain their decolorization mechanism. Thus, the strain *S. pavanii* GXUN74707 has a wide potential application, high adsorption efficiency, and stable performance, and is cost-effective for the treatment of dye wastewater, thereby promoting the development of dye wastewater treatment technology toward efficiency, adaptability, economy, and cleanliness.

Microbial flocculants are mainly studied in the laboratory. These experiments must be scaled up to evaluate the feasibility of utilizing microbial flocculants in large-scale industrial applications. The industrial use of bioflocculants depends on their long-term stability. The stability and reusability of bioflocculants require further research (Fazila et al., 2021; Manikant et al., 2023). Next, we can use bioinformatics to combine all omics data with genetic engineering data to comprehensively understand the microbial flocculation process. In addition, the processing capacity of individual strains is limited, and we need to understand and create synthetic microbial communities in order for microorganisms to survive in various harsh environments and exert flocculation functions (Babita and Pratyoosh, 2020; Shashi et al., 2022).

## Data availability statement

The original contributions presented in this study are included in this article/supplementary material, further inquiries can be directed to the corresponding authors.

## Author contributions

SQ: Conceptualization, Data curation, Formal analysis, Methodology, Project administration, Validation, Writing – original draft, Writing – review & editing. QL: Data curation, Formal analysis, Methodology, Writing – original draft. JD: Data curation, Methodology, Writing – original draft. YM: Data curation, Methodology, Writing – original draft. LW: Data curation, Methodology, Writing – original draft. HT: Data curation, Methodology, Writing – original draft. MJ: Writing – original draft, Writing – review & editing. GL: Formal analysis, Funding acquisition, Project administration, Resources, Writing – original draft, Writing – review & editing.
